# Implications of guppy (*Poecilia reticulata*) life‐history phenotype for mosquito control

**DOI:** 10.1002/ece3.2666

**Published:** 2017-04-01

**Authors:** Misha L. Warbanski, Piata Marques, Therese C. Frauendorf, Dawn A. T. Phillip, Rana W. El‐Sabaawi

**Affiliations:** ^1^ Biology DepartmentUniversity of VictoriaVictoriaBCCanada; ^2^ Department of Life SciencesThe University of the West IndiesSt AugustineTrinidad and Tobago

**Keywords:** biological vector control, Dengue, invasive species, mosquito‐transmitted disease, Zika

## Abstract

Guppies (*Poecilia reticulata*) are frequently introduced to both natural and artificial water bodies as a mosquito control. Laboratory studies have demonstrated that guppies can consume large numbers of larval mosquitoes. Our study investigates how intraspecific variability in guppy phenotype affects their importance as a mosquito biocontrol and how habitat conditions (natural ponds vs. water storage containers) may influence insect biomass and guppy feeding. Using a blocked experimental design, we established stream‐side mesocosm ponds with half receiving gravel substrate to simulate pond‐bottom habitat. To provide realistic diet choices and insect abundances, we allowed the mesocosms to colonize naturally with aquatic insect larvae for 1 month before introducing guppies. We tested two distinct guppy phenotypes (from high‐ and low‐predation streams) alongside fish‐free controls. After 1 month, we measured insect biomass in the mesocosms and examined guppy gut contents to document direct predation. While overall insect biomass was not significantly different across the three fish treatments, we observed a significant reduction in mosquito biomass in fish treatments compared to fish‐free controls, as well as intraspecific differences in feeding. Overall insect biomass was significantly higher in mesocosms without gravel, while habitat condition had no effect on mosquito biomass. As guppy phenotype responds to changes in their environments, it is an important consideration for biocontrol policy to anticipate potential ecosystem effects. We close by relating our findings to other studies and by discussing the implications and potential risks of using guppies to control mosquitoes.

## 1 Introduction

Mosquito‐transmitted disease prevention is a global priority. Importantly, controlling mosquito populations can reduce transmission of illnesses including dengue fever, malaria, chikungunya, and Zika. Even in regions with low incidence of disease, biting mosquitoes are considered a nuisance and both individuals and governments take action to prevent bites and control populations. The combined effects of climate change, urbanization, and water/waste management are credited with the proliferation of mosquito‐borne disease to new regions, and outbreaks are becoming more frequent (World Health Organization, 2014). Most recently, the Zika virus in Brazil (Campos, Bandeira, & Sardi, 2015) has made international headlines over confirmed links to birth defects (Rasmussen, Jamieson, Honein, & Petersen, 2016). Although new vaccines to prevent dengue fever and chikungunya are in varying stages of testing and approval (Capeding et al., 2014; Ramsauer et al., 2015), the main preventive strategy is to avoid mosquito bites (Centres for Disease Control and Prevention).

While chemical pesticides present a main line of defense in mosquito control, concerns over their evolutionary and ecological impacts (Guyton et al., 2015; Pimsamarn, Sornpeng, Akksilp, Paepornand, & Limpawitthayakul, 2009; Stanczyk, Brookfield, Ignell, Logan, & Field, 2010) lead to consideration of other options. One such option is to introduce predatory fishes to feed on mosquito larvae, because mosquitoes are known to lay their eggs in or near sources of standing water, and their larval stage is aquatic. Historically, guppy fish (*Poecilia reticulata*) have commonly been used (Deacon, Ramnarine, & Magurran, 2011), and are still deployed today into artificial ponds, water storage containers, sewers, and natural waterways (Deacon et al., 2011; News report, 2013, 2016; Pyke, 2008; World Health Organization).

Trinidadian guppies are a widely studied model of ecology and evolution. High‐profile introductions of guppies into previously guppy‐free and predator‐free areas have shown rapid evolution in response to specific invasion and predator release (Reznick, Butler, & Rodd, 2001). A suite of changes in guppy phenotype (i.e., morphology and life history) corresponds to changes in predatory regime, yielding distinct high‐predation (HP) and low‐predation (LP) phenotypes (Reznick et al., 2001). Guppy phenotype tends to be sensitive to short‐term growth conditions and long‐term adaptive history (Magurran, 2005) and can significantly alter guppy feeding patterns and preferences (Zandonà et al., 2011). Diet appears to be correlated with life‐history phenotype (Bassar et al., 2010; Palkovacs, Wasserman, & Kinnison, 2011; Zandonà et al., 2011). This implies that the efficacy of insect feeding in guppies is sensitive to the guppy's evolutionary history and its phenotype. Until now, this aspect of guppy ecology has been absent from mosquito control literature. Native guppy range includes the Caribbean islands and the northeast coast of South America (Magurran, 2005). However, due to its prevalence in the aquarium trade, and widespread use for mosquito control, the small fish has been introduced to many environments outside of its native range and has established populations in 60 countries globally (Deacon et al., 2011). Guppies are considered invasive and may cause impacts on both aquatic communities and ecosystem processes (Fraser & Lamphere, 2013; Holitzki, MacKenzie, Wiegner, & McDermid, 2013).

Biological management of mosquitoes relies on diet and behavior assumptions that predators, in this case guppies, will consume sufficient larvae to reduce insect emergence. Numerous studies have found guppies to be effective predators of mosquito larvae (e.g., Elias, Saidul Islam, Humayun Kabir, & Khalilur Rahman, 1995; Saleeza, Norma‐Rashid, & Sofian‐Azirun, 2014; Seng et al., 2008; Wijesinghe, Wickramasinghe, Kusumawathie, Jayasooriya, & De Silva, 2009), and the World Health Organization lists guppies as a common choice (World Health Organization). However, the literature on their efficacy in mosquito control is equivocal, with differing experimental design yielding different conclusions (Kusumanwathie, Wickremasinghe, Karunaweera, & Wijeyaratne, 2008), and some studies conclude guppies have little to no demonstrable benefit (Dua, Pandey, Rai, & Dash, 2007; Lawal, Edokpayi, & Osibona, 2012). Contradicting conclusions make it difficult to weigh the costs and benefits of using guppies to balance the risk of introduction against perceived benefit to public health.

The literature on mosquito management has not yet integrated ecological and evolutionary research on guppies. Our study is motivated by a desire to reconcile the too‐often disparate fields of theoretical and applied biology. As biocontrol management involves introducing guppies to novel ecosystems, and evolution of distinct life histories can occur rapidly, we wondered whether the effect of phenotype should be an important consideration for managers.

In this study, we conducted a mesocosm experiment to test the effect of guppies and guppy phenotype on mosquito populations. We asked how phenotypic differences in guppies affect diet preference, specifically consumption of larval mosquitoes. We hypothesized that larval insect biomass would be lower in mesocosm ponds with guppies compared to fish‐free controls, and expected to observe phenotypic differences in feeding between HP and LP guppies. Currently, competing hypotheses on guppy diet predict opposite outcomes. HP guppies may be specialist feeders adapted to eat high‐quality insect prey, while LP fish are generalist feeders (Bassar et al., 2010). Following this hypothesis, HP guppies would consume more insects than LP guppies. Alternatively, LP guppies reach higher densities in the wild and face greater intraspecific competition for resources and may become more efficient feeders than HP guppies (Palkovacs et al., 2011). Following this hypothesis, LP guppies would consume more food items in general, including more insects, than HP guppies.

We compared the effect of guppy presence and guppy phenotype in treatments with and without gravel substrate to test whether habitat characteristics play an important role in shaping aquatic insect communities and guppy foraging. Many previous studies are inconclusive and appear to depend on experimental setup and guppy treatment. This designed habitat difference also reflects the ways guppies can be kept for mosquito control: in artificial ponds with some habitat complexity or in residential and community water storage containers. Habitat features, including bottom substrate, temperature, and light, are considered an important factor affecting mosquitoes (Vezzani, Rubio, Velázquez, Schweigmann, & Wiegand, 2005) and other aquatic invertebrates (Voshell, 2002). We hypothesized that insect communities, and guppy diet would respond to habitat differences.

Finally, we measured ecosystem parameters (chlorophyll‐a, algal ash‐free dry mass, and ammonium concentrations) in the mesocosms because HP and LP guppies have been shown to affect not only their prey, but also the surrounding ecosystem, and because changes in insect composition might lead to broader ecosystem effects (Bassar et al., 2010; Holitzki et al., 2013). Previous experiments have shown that guppies consume algae and excrete nutrients (Bassar et al., 2010).

## 2 Methods

### 2.1 Mesocosm feeding experiment

**Figure 1:**
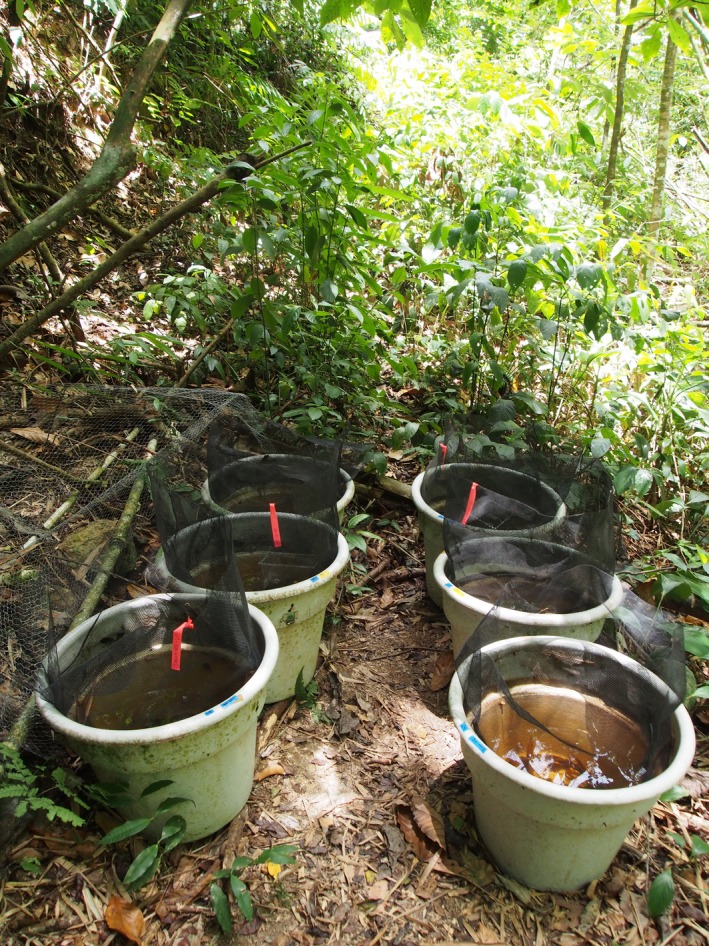
An experimental block in situ. One of four experimental blocks, containing six mesocosms situated on the bank of St. Patrick's River, Verdant Vale, Trinidad

We arranged 24 large white horticultural pots (described in Fraser & Lamphere, 2013) in four experimental blocks on the banks of St. Patrick's River in Verdant Vale, Trinidad (Figure 1). Each container was filled with filtered (64‐μm sieve) stream water to 35 cm depth. Half of the containers in each block received a 4‐cm‐deep layer of prewashed gravel substrate, while the other half had no substrate added.

The experiment included two phases: insect colonization and guppy feeding. During the insect colonization phase, the pots stood uncovered for 1 month to allow insects to establish populations. Leaves falling into the pots were removed from the water surface every 3 days to prevent buildup of organic matter and anoxic conditions. Before removal, leaves were lightly scrubbed to dislodge any attached eggs and invertebrates. Water quality (temperature, conductivity, and dissolved oxygen) was monitored at least twice weekly during this period using a multiprobe (YSI, Pro 2030 model). Water levels were monitored and filtered stream water was added as needed to maintain the standard depth. Overflow holes covered with 0.5 mm mesh allowed excessive rainwater to escape, but prevent guppies from being washed out.

Guppies were collected from pools at two sites within the Aripo drainage. The high‐predation (HP) site was within the main stem of the Aripo River (GPS: 10°66′56.8′′N, 61°22′78.9′′W), while the low‐predation (LP) site was situated above a barrier waterfall (GPS: 10°69′04.8′′N 61°23′68.9′′W) that excludes most of the major guppy predators including pike cichlids (*Crenicichla frenata*) and wolf fish (*Hoplias malabaricus*) (Haskins, Haskins, McLaughlin, & Hewitt, 1961; Reznick & Endler, 1982). The guppies from these populations exhibit the classic HP vs. LP phenotypic differences in life history, diet, and ecosystem effects and have been used in numerous experiments (Bassar et al., 2010; El‐Sabaawi et al., 2015; Palkovacs et al., 2011; Zandonà et al., 2011). In the laboratory, guppies were placed in 10‐L aquaria according to phenotype and sex. They were fed a diet of commercial flake food once daily. Guppies were monitored for 48 hr for health and signs of stress before they were introduced to the mesocosms.

**Figure 2:**
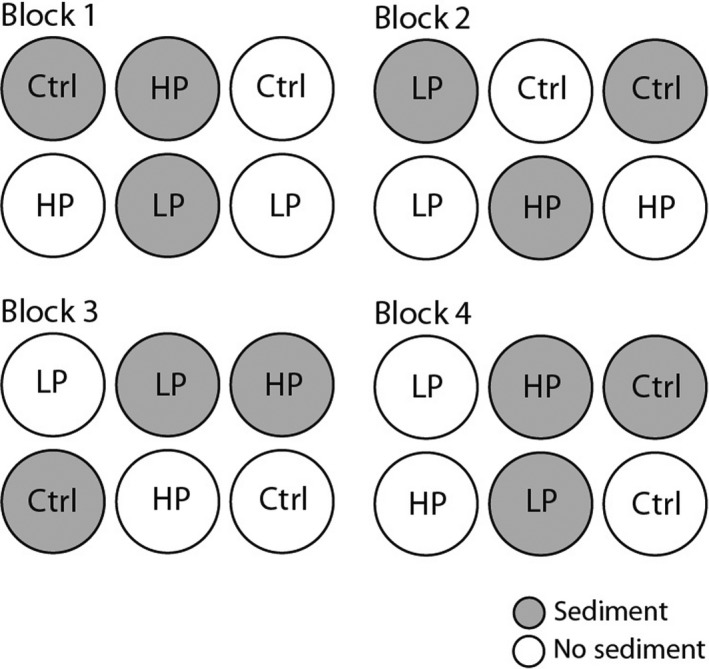
Experimental block design. Four experimental treatment blocks were established. Pots in each block were randomly assigned two sediment treatments: gravel (gray circles) and no gravel (white circles) and one of three fish treatments: high‐predation (HP), low‐predation (LP) or no‐fish control (Ctrl)

Fish treatments were randomly assigned within each block (Figure 2). We introduced HP and LP guppies to experimental pots in equal numbers, according to population. Each guppy‐assigned pot received two male and two female fish, while two pots in each block were guppy‐free controls. To minimize disturbance to the fish from piscivorous birds and snakes, chicken wire (2.5 cm mesh) was fitted over each pot. This mesh size was large enough to allow aquatic invertebrates to continue colonization, and not significantly block daylight penetration into each pot.

Guppies were monitored daily for the duration of the month‐long feeding phase, which is sufficient to observe ecosystem effects from guppies (El‐Sabaawi et al., 2015). Any deceased fish were removed and replaced with healthy fish to ensure a constant number of fish feeding in each mesocosm. Water quality (dissolved oxygen, conductivity, and temperature) was measured weekly. Temperature variation between experimental blocks was 0.1°C (Table S1, *p* < .001) (mean for blocks 1, 2, and 3 was 22.7°C, and 22.8°C for block 4) and was not influenced by gravel treatment (Table S1, *p* = .458). Oxygen levels remained sufficiently high throughout the experiment (Weber & Kramer, 1983); no anoxic events were observed. Percent canopy cover over experimental blocks, measured with a concave spherical densitometer, was similar across blocks (Table S2, *p* = .856) ranging from 94.4% to 96.4% canopy.

At the end of the experimental period, we removed the guppies from the mesocosms and measured ecosystem parameters. We euthanized the guppies with an overdose of MS‐222 and measured standard length and mass before preserving them in formalin for gut content analysis. Water samples were collected and analyzed for ammonium (Holmes, Aminot, Kerouel, Hooker, & Peterson, 1999; Taylor et al., 2007). Attached algae was collected from the sides of the mesocosms using a Loeb sampler (Loeb, 1981); chlorophyll‐a (a proxy for the photosynthetically active part of algae) was extracted from a subsample from each mesocosm with 95% ethanol and the concentration measured using fluorescence (see Arar & Collins, 1997). Algal ash‐free dry mass (AFDM) (a measure of algal quantity) was determined by filtering a subsample of the slurry onto pre‐ashed glass‐fiber filters. The filters were dried, weighed, combusted in a muffle furnace at 500°C, and weighed again to correct for inorganic material.

To collect insects, bottom sediments (when present) were agitated to disturb benthic invertebrates and the water from each mesocosm was filtered through 250‐μm sieves. The sides of the mesocosms and any leaf matter were rinsed with filtered water to dislodge insects. The collected insects were preserved in 70% ethanol.

### 2.2 Insect identification and biomass estimates

In the laboratory, insects were separated from organic material, identified to genus whenever possible (using Heatherly, 2012; Merritt, Cummins, & Berg, 2008; University of Alberta; Voshell, 2002), and counted to calculate total abundance per pot. We estimated insect biomass using our own length–mass relationships for 14 taxa (S2 Appendix) that together made up 98% of overall insect abundance. We computed power relationships for length and mass using methods adapted from Benke, Huryn, Smock, & Wallace (1999). In each sample, we measured the standard length of the first 100 specimens of each taxon to the nearest millimeter against 1‐mm grid paper. For each taxon, we subsampled insects across their size range (~20–40 individuals) to be photographed alongside a scale bar using a Leica M420 macroscope, scale bar, and a Spot digital camera. Insects were placed in pre‐ashed foil weighing boats and dried at 55°C for 24 hr, cooled, and weighed to the nearest 0.001 mg dry mass (DM). Small insects (3 mm or less) were pooled in groups of at least three similar‐sized individuals, and an average length and mass were computed. To correct for inorganic materials in the intestinal tract, dried insects were then ashed in a muffle furnace for 1 hr (450°C) and weighed again to determine insect AFDM. We used insect AFDM values to calculate biomass. We computed diversity metrics for each mesocosm: species richness, evenness, and the Shannon–Wiener diversity index.

### 2.3 Gut contents

Fish guts were removed, measured for length to the nearest mm, and dissected to examine contents. Insect head capsules were counted and identified to family. Head capsules are generally made up of heavily sclerotized exoskeleton and as a result are resistant to digestion (Hopkins & Kramer, 1992). The entire gut was analyzed in order to minimize zero‐counts.

### 2.4 Statistical analysis

We modeled insect abundance and biomass, using linear mixed effects models using the lme4 package in R version 3.1.3 (Bates, Maechler, Bolker, & Walker, 2015) for the following response variables: overall insect community, mosquitoes (Culicidae), midges (Chironomidae), and mayflies (Ephemeroptera), measured as milligrams of biomass. We singled out midges because they are considered to be an important food item for guppies collected from natural environments (Manna, Aditya, & Banerjee, 2008; Zandonà et al., 2011), and because they were highly abundant in our mesocosms. We included mayflies because they are abundant in natural stream ecosystems and occasionally observed in guppy gut contents (Zandonà et al., 2011). Fish treatment (no fish, HP, and LP) and substrate treatment (gravel/no gravel) were included in the models as fixed effects, while experimental block number was included as a categorical random effect.

Interactions between the guppy treatment and the gravel treatment were included in initial model runs. Full models, including interactions, were compared with a model that omitted the interaction using the Akaike information criterion (AICc). If there was no significant difference between the models, the interaction was dropped. Pairwise contrasts between groups were tested through generalized linear hypothesis tests using a Tukey HSD method that corrects for multiple comparisons. Residuals were examined using Q–Q plots. Total insect biomass, mosquito biomass, and abundance and mayfly biomass and abundance data were log+1 transformed to improve model fit. Mosquito data from one control mesocosm were omitted as an outlier. It held 41.65 mg of mosquito AFDM compared to the average of 2.55 mg in other like treatments.

We followed the same linear modeling approach for insect diversity measurements as well as the ecosystem response variables: chlorophyll‐a concentration, algal AFDM, and NH_4_ concentration.

We analyzed differences in the gut contents in two ways: once using insect counts (defined as the number of mosquitoes in all guts counted) and once on proportions (defined as the proportion of mosquitoes in all guts counted). For insect counts, Fisher's exact test was used to evaluate differences between HP and LP guts for overall insects, mosquitoes, and chironomids. This test allows for gut item counts of less than five.

Next, for each experimental block, we computed the proportion of fish guts containing insect prey. Using these proportions as a response variable, we used a two‐way ANOVA (with fish phenotype and sex as categorical variables) to test for differences between groups. We included sex as a categorical variable because previous studies reported differences in feeding between the sexes (Magurran, 2005).

### 2.5 Animal care and ethics

Our experiment conformed to the animal care and ethics guidelines of the Canadian Council for Animal Care (CCAC) (University of Victoria Animal Use Protocol # 2014‐004).

## 3 Results

### 3.1 General ecosystem effects

While guppies had no effect on chlorophyll and ammonium, gravel substrate had only a small effect (Tables S3 and S4). We observed slightly higher chlorophyll‐a concentrations (*p* = .057) in treatments with gravel, but no difference in ammonium concentrations (*p* = .110).

### 3.2 Insect diversity and biomass

Overall, we identified 39 distinct aquatic and semi‐aquatic insect taxa, across nine orders in the mesocosm ponds. Midges (Chironomidae), mosquitoes (Culicidae), and mayflies (Caenidae and Leptophlebiidae) were most abundant. Within the mosquito family, six genera colonized the mesocosms: *Culex*,* Anopheles*,* Culiseta*,* Wyeomyia*,* Haemagogus*, and a single individual of the predaceous *Toxorhynchites*. The genus *Aedes*, the common disease vector for the dengue, chikungunya, and Zika viruses, was notably absent.

Both fish and substrate treatments had significant effects on insect species richness (Table 1, *p* = .024; *p* = .034). The presence of guppies led to a 30% reduction in overall insect richness compared to controls, but there were no significant differences in insect richness between guppy phenotypes (Tables 1, S6 and S7, *p* = .988). Insect richness was 20% higher in mesocosms with gravel substrate than those without (Table S5), and the interaction between the fish treatment and the gravel treatment was not significant.

**Table 1:** The effects of fish (no‐fish control, high predation (HP), low predation (LP)) and substrate (gravel, no gravel) on ecosystem response variables

Variable	Fish	Substrate
Richness	8.81_2,17_ **	5.29_1,17_ *
Diversity	3.19_2,17_$	0.33_1,17_
Evenness	0.53_2,17_	2.47_1,17_
Insect biomass	1.18_2,17_	10.46_1,17_ *
Midge biomass	1.31_2,17_	16.14_1,17_ ***
Mosquito biomass	57.13_2,16_ ***	0.62_1,16_
Mayfly biomass	0.63_2,17_	0.71_1,17_

Main entries are *F*‐ratios; degrees of freedom are listed in subscript. These values were obtained from linear mixed modeling as outlined in the text. Statistical significance is noted at the following levels: $ is *p* < .10, * is *p* < .05, ** is *p* < .01, and *** is *p* < .001. Diversity is Shannon–Wiener diversity index.

Shannon–Wiener diversity, which adjusts species richness with the relative abundance of individuals, was marginally higher in controls than in fish treatments (Table 1, *p* = .067), but was not affected by gravel. (Table 1, *p* = .576). The Shannon–Wiener diversity index was 1.14 in controls and 0.89 in both HP and LP treatments (Table S5). There were no differences in diversity between guppy phenotypes (*p* = .999). Neither fish treatment nor substrate had a significant effect on evenness (the distribution of individuals among species) (Table 1, fish treatment: *p* = .596, substrate: *p* = .135).

**Figure 3:**
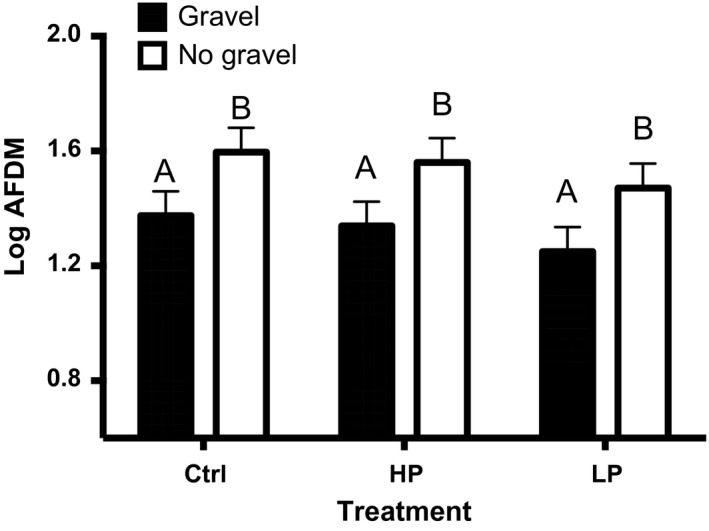
Insect biomass Least‐square means (+ standard error) by fish and substrate treatment for total insect biomass, expressed as log(ash‐free dry mass). Shaded bars show gravel treatment, while white represents replicates without gravel. Ctrl represents fish‐free controls, and HP and LP represent replicates with fish from high‐predation and low‐predation stream environments, respectively (*n* = 24). Statistically significant differences are indicated by different letters above bars

Total insect biomass was not affected by the presence of fish, but was instead more strongly determined by the presence of bottom substrate (Figure 3, Table 1, fish treatment: *p* = .330, substrate: *p* = .005). Average total insect biomass was highest in control mesocosms (gravel: 25.8 mg, no gravel: 44.4 mg), followed by HP treatments (gravel: 20.8 mg, no gravel: 42.1 mg) and LP treatments (gravel: 19.6 mg, no gravel: 32.1 mg) (raw means: Table S6; marginal means: Table S7).

**Figure 4:**
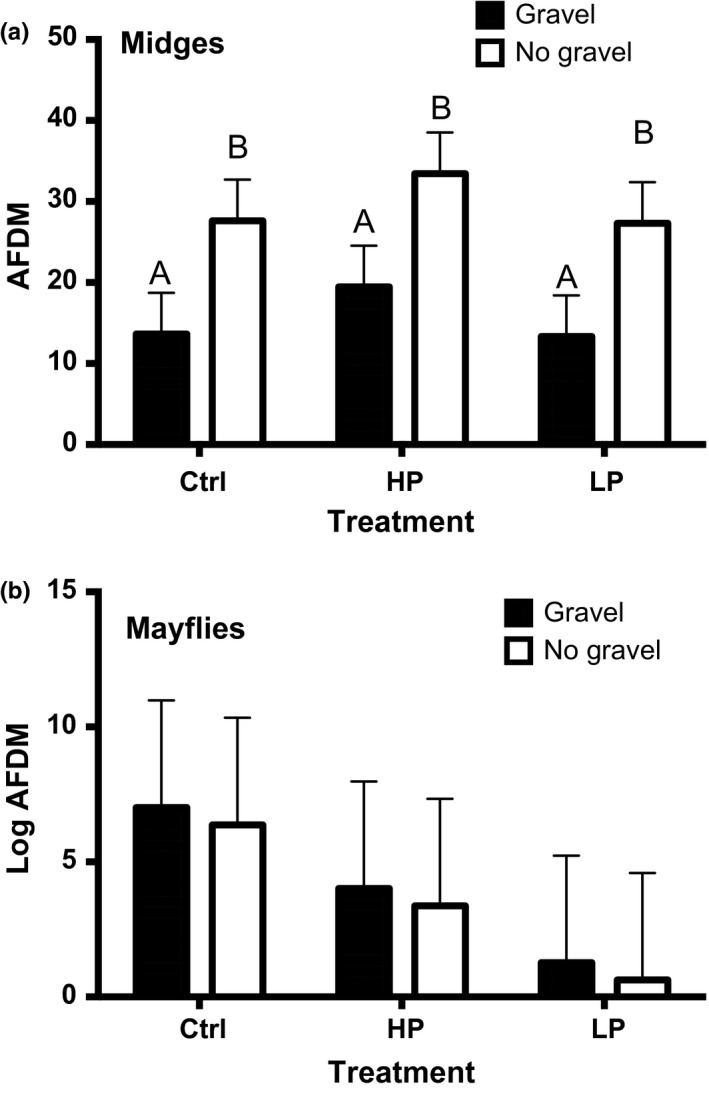
Midge and mayfly biomass. Least‐square means (+ standard error) by fish and substrate treatment for (a) midge biomass (*n* = 24) and (b) mayfly biomass (*n* = 24). Biomass is expressed as log(ash‐free dry mass). Shaded bars show gravel treatment, while white represents replicates without gravel. Ctrl represents fish‐free controls, and HP and LP represent replicates with fish from high‐predation and low‐predation fish stream environments, respectively. Statistically significant differences are indicated by different letters above bars

Midges were the most dominant insect group to colonize the mesocosms, representing between ~65% and 85% of insect biomass across mesocosms. Midge biomass followed a similar pattern to the overall insect biomass (Figure 4). Midge biomass was affected more by substrate than by fish presence and phenotype (Table 1, *p* < .001 and *p* = .296, respectively). Midge biomass in mesocosms with no gravel substrate (Table S6, control: 28.9 mg, HP 35.4 mg, LP: 24.1 mg) was approximately double than those with gravel (Table S6, control: 12.3, HP: 17.6, LP: 16.5 mg). The highest midge biomass was associated with HP fish treatments, but the trend was not significant (Table S7).

Mayflies made up ~4%–16% of insect biomass in the mesocosms. Biomass was highest in controls (Table S6, gravel: 10.0 mg, no gravel: 2.2 mg), followed by HP (gravel: 0.2 mg, no gravel: 6.2 mg) and LP mesocosms (gravel: 1.0 mg, no gravel: 0.6 mg). In contrast to the strong effect of guppy presence on mosquitoes, mayfly biomass was not affected (Figure 4, Tables 1 and S7, *p* = .546). Gravel substrate also did not significantly affect mayfly biomass (Tables 1 and S7, *p* = .412).

**Figure 5:**
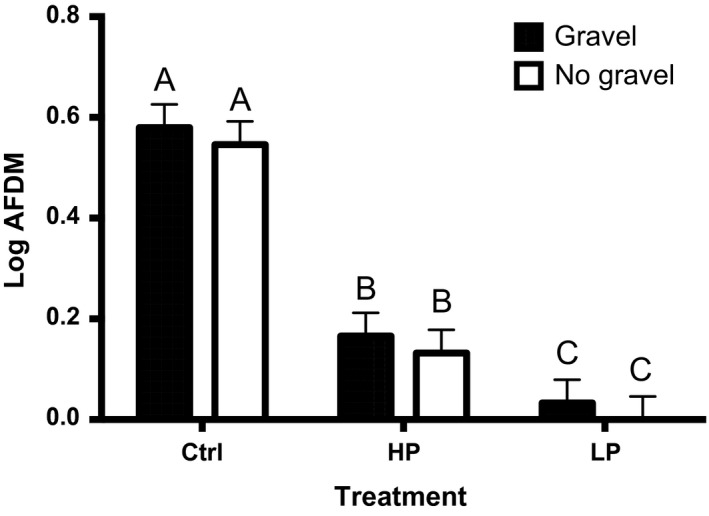
Mosquito biomass. Least‐square means (+ standard error) by fish and substrate treatment for mosquito biomass, expressed as log(ash‐free dry mass). Shaded bars show gravel treatment, while white represents replicates without gravel. Ctrl represents fish‐free controls, and HP and LP represent replicates with fish from high‐predation and low‐predation fish stream environments, respectively (*n* = 23). Different letters above bars show statistically significant differences between treatments

Mosquitoes, on average, represented ~17% of insect biomass across control mesocosms, but only 1.6% in HP mesocosms and 0.1% in LP mesocosms. Guppy presence had a significant negative effect on mosquito biomass (Figure 5, Tables 1 and S7, *p* < .001). But mosquito biomass was not affected by the substrate treatment (Table 1, *p* = .443). Average mosquito biomass was an order of magnitude higher in controls (Table S6, gravel: 3.1 mg, no gravel: 2.6 mg) than HP guppy mesocosms (gravel: 0.5 mg, no gravel: 0.4 mg) and two orders of magnitude higher than LP guppy mesocosms (gravel: 0.04 mg, no gravel: 0.04 mg). LP treatments contained significantly fewer mosquitoes than HP treatments (*p* = .013). Despite this statistical difference, both phenotypes reduced larval mosquito biomass by over 99% compared to controls. Total insect, midge, and mayfly abundance were not affected by either fish treatment or substrate treatment; however, mosquito abundance was significantly lower where fish were present (see Tables S3, S6, and S7). Mosquito larvae collected from fish‐containing mesocosms measured 5 mm or smaller, whereas those from control mesocosm reached up to 10 mm length.

### 3.3 Gut contents

**Figure 6:**
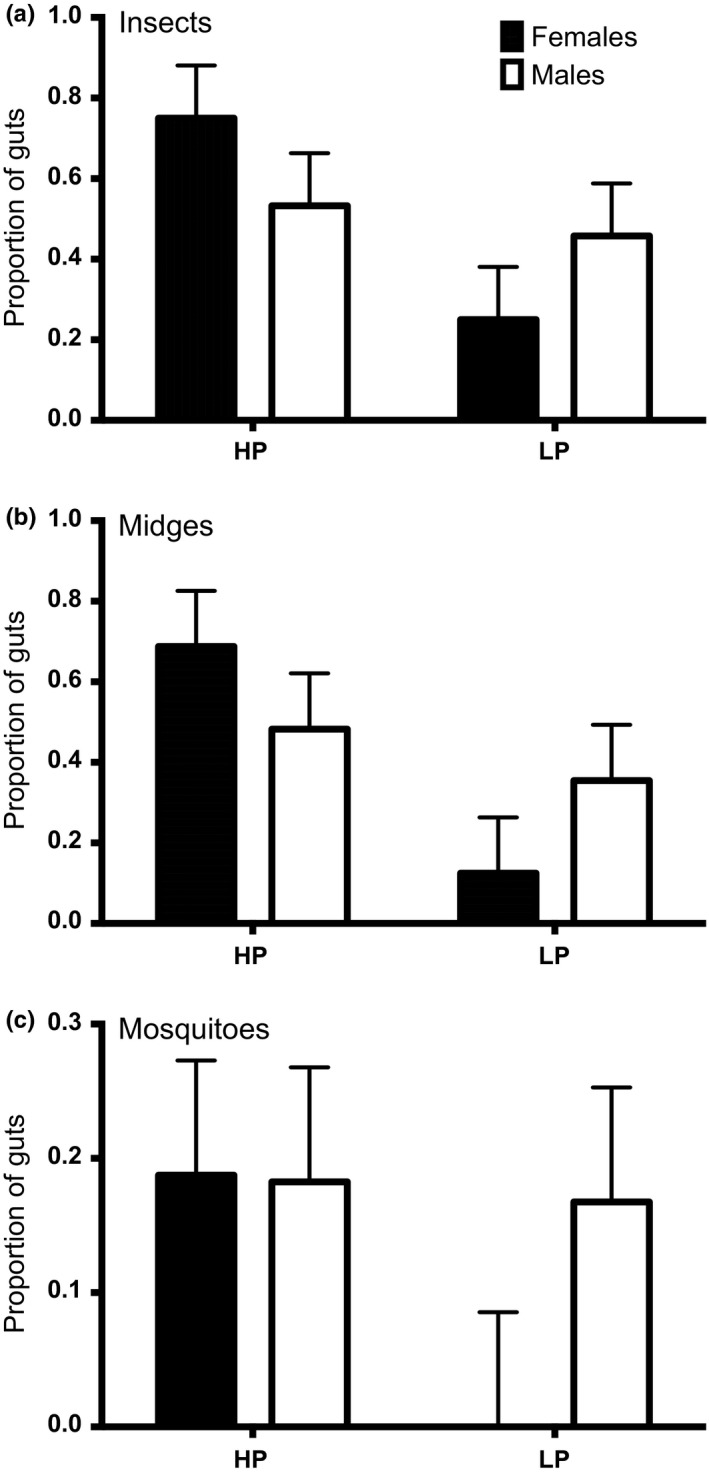
Guppy diet analysis. The mean proportion (+ standard error) of guts containing (a) insects, (b) midges, and (c) mosquitoes by HP and LP phenotypes (*n* = 16). No LP females examined consumed mosquitoes (panel c)

While the overall insect biomass data showed no difference between guppy phenotypes, our gut content analysis showed that insect feeding was more prevalent in HP guppies than LP guppies. More than half (64.3%) of HP guppies had insects in their guts, compared to 35.5% of LP guppies (Figure 6), a statistically significant difference (Table S8, *p* = .038, odds ratio=3.204). Although mosquitoes and midges were prominent diet items, we found no significant differences between phenotypes for either midges or mosquito consumption (Figure 6), suggesting that both HP and LP guppies had an equal tendency for feeding on mosquitoes.

Total insect prey items per guppy ranged from 0 to 14; midges and mosquitoes were most common. Only a single mayfly was observed in the guts. Omitting one outlying individual (an HP guppy that had consumed 14 midge larvae), the average gut with insects present contained 2.3 ± 1.5 and 2.2 ± 1.5 prey items for HP and LP fish, respectively. In both populations, female fish were significantly larger than male fish (*p* < .001), and LP fish were larger than HP fish (*p* = .010). Average standard length was 16.3 mm for HP males and 19.1 mm for HP females. Average standard length of LP males was 17.1 mm and 20.5 mm for LP females. The effects of sex were generally minor and varied slightly between phenotypes (Table 2, *p* = .048). Within HP guppies, females consumed slightly more insect prey than males, but the reverse was true for LP guppies. None of the LP females examined had consumed mosquito larvae. The differences in diet were driven by female guppies (Tukey's HSD, *p* = .078). In contrast to the mosquito abundance data, gut contents did not show differences in mosquito consumption between guppy phenotypes (Table 2, *p* = .258).

**Table 2:** Results of a two‐way ANOVA on the proportion of guppy guts containing insect prey, midges, and mosquitoes

Variable	Phenotype	Sex	Phenotype^a^sex
Insect prey	4.843_1,12_ a	0.001_1,12_	2.646_1,12_
Midge prey	6.229_1,12_ a	0.008_1,12_	2.476_1,12_
Mosquito prey	1.409_1,15_	0.907_1,12_	1.022_1,12_

Main entries are F‐ratios; degrees of freedom are listed in subscript.

a represents statistical significance of *p* < .05.

## 4 Discussion

The purpose of this study was to test the effect of guppy presence on aquatic invertebrates in experimental ponds, with particular attention to larval mosquitoes. We also asked whether phenotypic variation in guppies affects their diet of invertebrates in general and mosquitoes in particular. Finally, we asked whether simple differences in habitat structure have an effect on the invertebrate community.

Guppies significantly reduced larval mosquito biomass in the mesocosms compared to controls. Gut content analysis confirmed mosquito consumption by both high‐ (HP) and low‐predation (LP) guppy phenotypes, and insect community analysis found both phenotypes reduced larval mosquito biomass by more than 99% compared to controls. Guppy presence was associated with significantly fewer insect taxa and lower Shannon–Wiener diversity than fish‐free controls. However, overall insect biomass was not significantly different between guppy and guppy‐free treatments.

While guppy presence significantly depressed insect diversity, richness, and mosquito biomass, differences between the HP and LP phenotypes were most pronounced for their effect on larval mosquitoes. We found that LP guppies consumed significantly more mosquitoes than their HP counterparts. Mosquito biomass was an order of magnitude lower in HP mesocosms and two orders of magnitude lower in LP mesocosms than controls.

Fish gut contents suggest greater insect consumption among HP than LP fish, driven in particular by the diets of female fish. However, no difference in guppy diet was detected between phenotypes for larval mosquito consumption.

Total insect biomass and midge biomass were both sensitive to bottom substrate treatment. Total insect biomass was significantly lower in gravel‐containing treatments, but species richness was higher. Midges were doubly abundant in treatments without gravel. Substrate had no significant effect on mosquito biomass.

### 4.1 The role of guppy phenotype

Our results suggest that guppies, regardless of phenotype, can effectively reduce larval mosquito populations in water storage containers and artificial ponds. The 99% reduction in mosquito biomass is consistent with Wijesinghe et al., (2009) who achieved a 90%–100% reduction in mosquitoes by introducing between one and three fish to concrete household water storage tanks.

Observations of wild guppies show distinct phenotypes, with predictable differences in diet (Reznick et al., 2001). These patterns hold under common garden experiments (Bassar et al., 2010). However, two competing hypotheses have emerged to explain the patterns. The first argues that HP fish are specialist feeders adapted to eat insect prey, while LP fish are generalist feeders (Bassar et al., 2010). The second posits LP guppies are more efficient feeders due to the effects of predator release, increased guppy density, and heightened resource competition, meaning that they capture and potentially deplete food resources more efficiently (Palkovacs et al., 2011). Our insect community results provide support for the second hypothesis that LP guppies are the more efficient feeders because they had a stronger negative effect on mosquito larvae than HP fish. Gut content results suggest HP fish feed more on insects, in particular midges, than LP fish, which seems to support the first specialist feeding hypothesis, but contradicts the conclusions drawn from insect community data that showed no significant differences in total insect biomass among HP, LP, and control mesocosms. It is possible that in our mesocosms, where insects were abundant and predators few, the preference for insects by HP fish did not have a significant effect on the insect community or ecosystem. Therefore, our results support both hypotheses, showing that they may not be mutually exclusive from each other. Differences between studies may be due to how feeding was evaluated (from guts, vs. insects present in the environment) or differences in insect availability. Recent studies also show that there is considerable plasticity in guppy feeding especially across seasons (Zandonà, Auer, & Kilham, 2015; Zandonà et al., 2011) further supporting a role for resource availability in determining guppy diet.

The combination of gut content analysis and resource availability in the ecosystem provides context to help interpret results. It is possible that LP guppies depleted mosquitoes earlier in our experiment and then moved on to other items. We suggest that a time series approach to better understand how fish diet and the insect community change together after introduction may also be helpful for future studies.

### 4.2 Comparison with other studies

Studies of guppy diet in Trinidad rarely mention the consumption of mosquitoes, either because they were absent from the experimental mesocosms used in these studies (El‐Sabaawi et al., 2015), or because they may be grouped with other dipterans in gut content analysis (Zandonà et al., 2011). The second study suggested that midges (family Chironomidae) are a preferred food item, representing about 40% of a guppy's insect diet, while other dipterans make up at 13%. Caddisflies (order Trichoperta) made up 14%, while mayflies (order Ephemeroptera) were generally avoided by HP guppies and made up 8% overall (Zandonà et al., 2011). One possibility to explain the absence of mosquitoes is that these studies all take place in streams and rivers, or in experiments with moving water, and mosquitoes are known to prefer laying their eggs in sluggish/stagnant waters.

The body of the literature on Trinidadian guppies reveals that guppies can have strong effects on their environments and that guppy phenotype can be an important factor (Bassar et al., 2010; Palkovacs et al., 2011). In contrast to previous experiments, which reported that guppy phenotype had a significant effect on chlorophyll‐a and ammonium (Bassar et al., 2010; Holitzki et al., 2013), we did not observe such differences. We propose that this is due to differences in experimental structures. For example, we observed that chlorophyll‐a and ammonium were more sensitive to the presence of gravel than to differences in guppy phenotypes, suggesting that they are indeed sensitive to design parameters. In natural systems, guppy density is also affected by predatory regime, with LP populations reaching higher densities than HP populations, and has been shown to be more important than phenotype for determining a variety of ecosystem measurements (Bassar et al., 2010). Our fish densities may have been too low to trigger any cascading effects.

### 4.3 The role of substrate

Mesocosms with gravel were designed to offer greater habitat complexity and to mimic some differences between natural pools and water storage containers. The bottom treatment had no statistically significant effect on either algal quality (chlorophyll‐a) or standing stocks (AFDM). However, mesocosms with gravel contained significantly fewer midges and insects overall, an outcome that was not expected. Temperature, oxygen, and conductivity, measured at the mid‐water level, were similar between substrate treatments. Sediment conditions (e.g., sorting and size) might explain these unexpected patterns; however, the larger species richness associated with gravel substrates suggests that sediment conditions were conducive to insect colonization. None of our models showed significant interactions between bottom substrate and guppy feeding, which we take to suggest that as visual predators, guppy foraging was not affected by bottom substrate. Our study shows that differences in habitat can be predictors of insect community trends (specifically midge biomass and abundance) and therefore highlights the context dependency of experimental outcomes.

### 4.4 Implications for management

Our results provide evidence that guppy introductions to artificial pools can reduce larval mosquito populations, regardless of either fish phenotype and or habitat complexity. Our study supports the use of guppies as larvacidal agents. However, further introductions for mosquito control should be cautiously considered.

While our study documented dramatically fewer mosquitoes in mesocosms containing guppies, as well as direct consumption by both phenotypes, mosquitoes are not commonly reported prey in other studies using the same populations (Aripo River) (Bassar et al., 2010; Zandonà et al., 2011, 2015). This suggests guppy diet is context dependent, despite repeatedly observed differences between HP and LP phenotypes. As a result, it is likely difficult for managers to predict outcomes and evaluate costs and benefits of introducing guppies for mosquito control.

Even if they are housed in water cisterns or artificial ponds, guppies may be discarded or escape into local ecosystems during floods. The costs of accidentally or intentionally introducing guppies to previously guppy‐free systems are staggering. In a comparison of guppy‐invaded and guppy‐free Hawaiian streams, Holitzki et al., (2013) found strong changes in ecosystem structure including increased carbon and nitrogen loading, increased benthic biofilm, as well an altered invertebrate community structure. Notably native goby abundance decreased twofold following guppy invasions (Holitzki et al., 2013). It is also unlikely that accidental or deliberate guppy introductions to local water habitats will result in an overall reduction in mosquito abundances. Guppies are often introduced into large aquatic watersheds or fast‐flowing rivers, which do not promote mosquito colonization.

Within their native range, guppies can alter ecosystem processes when they are introduced to previously guppy‐free streams by increasing primary production through nutrient excretion (Bassar et al., 2010), with the potential of altering dynamics downstream. Guppies also have a negative impact on Trinidad's native killifish (*Anablepsoides hartii*) populations (Fraser & Lamphere, 2013). Declines have been documented through controlled introductions of guppies to previously guppy‐free portions of streams. Laboratory and artificial stream studies that suggest both competition for resources and predation by guppies on larval killifish play a role in these declines (Fraser & Lamphere, 2013). A survey of 12 fish species in Sri Lanka found guppies were only marginally better at controlling mosquitoes than native fish alternatives (Kusumawathie, Wickremasinghe, Karunaweera, & Wijeryaratne, 2006). International guidelines recommend employing a native larvicidal species over exotic species whenever possible; however, even this carries risks.

The very characteristics that make guppies good candidates for introduction, such as live birth and sperm storage, also make them successful ecosystem invaders. Deacon et al. (2011) demonstrated that a single pregnant female can establish a viable population 86% of the time. Even if introduced to residential water containers, and artificial ponds, discarded fish or accidental release to the environment during heavy rains may still pose a threat to natural systems, even within native guppy range. Controlling the spread of mosquito‐borne diseases such as malaria and dengue fever remains a public health priority. But the impacts of fish introductions for this purpose must be carefully weighed against their benefits.

Our mesocosms attracted a variety of mosquito taxa, including several potential vector genera. *Anopheles*,* Culex*, and *Haemagogus* are known to transmit disease in certain geographic areas, including malaria, West Nile virus, and yellow fever (Cardoso et al., 2010; Centres for Disease Control and Prevention; World Health Organization). *Aedes*, a vector for Zika and dengue fever, was notably absent from our mesocosms despite a tendency to readily colonize a variety of man‐made habitats (Harrington, Ponlawat, Edman, Scott, & Vermeylen, 2008; Samson et al., 2015). It is thought that *Aedes* thrive in urban and suburban areas (Davis, Kline, & Kaufman, 2016; Tsuzuki, Huynh, Luu, Tsunoda, & Takagi, 2009), but it is unclear what threshold of urbanization promotes *Aedes* proliferation. It would be important to managers to additionally consider the habitat requirements of target mosquito vectors when designing control strategies.

In conclusion, our study finds that guppies, regardless of phenotype, can effectively reduce larval mosquito populations in isolated artificial ponds and/or water storage containers, although LP‐adapted fish are most effective. Considering life‐history phenotype might therefore offer an advantage for mosquito control in certain circumstances. The study also found that habitat features had little impact on mosquito biomass suggesting that placing guppies in separate artificial containers is just as effective as placing them in natural areas where the potential for invasion is high. Mosquito control programs that wish to use guppies should consider that guppies are efficient in controlling mosquito in small bodies of still water, such as water storage tanks or isolated artificial ponds, but little evidence in the literature suggests they perform better than some native species in natural bodies of water.

If a guppy‐based mosquito control program is preferred, explicit guidelines for guppy disposal and educational programs on the costs of invasion could help reduce introduction to the wild. If guppies are introduced to isolated water tanks and ponds, the risks of invasion might be well managed. However, if introduced in bigger bodies of water such as lakes, controlling naturalization may be impossible. When it comes to addressing mosquito‐borne illness, ecologically responsible management should consider both ecological and evolutionary consequences of the larvicidal agent, be it chemical or living. The application of basic science to public health policy should be used to find effective mosquito management solutions that work within the context of local geography without compromising ecological integrity.

## Conflict of Interest

None declared.
