# Supplementary material for: Implications of guppy (Poecilia reticulata) life‐history phenotype for mosquito control
Source: Ecol Evol. 2017 Apr 1;7(10):3324–34. doi: 10.1002/ece3.2666 (PMC5433973; doi:10.1002/ece3.2666)
Supplement: Supplementary file 1 [file ECE3-7-3324-s001.docx]

**S1 Appendix**

**S1 Table. Temperature variability among treatments**

| Source of Variation | *df* | Sum of Squares | Mean Squares | *F*-ratio | *P*-value |
| --- | --- | --- | --- | --- | --- |
| Substrate | 1 | 0.00167 | 0.00167 | 0.571 | 0.461 |
| Block | 3 | 0.10833 | 0.03611 | 12.381 | <0.001 |
| Substrate*Block | 3 | 0.00833 | 0.00278 | 0.952 | 0.439 |
| Error | 16 | 0.04667 | 0.00292 |  |  |
| Total | 23 | 0.165 | 0.04348 |  |  |

**S2 Table 2. Variability in percent canopy cover among blocks**

| Source of Variation | *df* | Sum of Squares | Mean Squares | *F*-ratio | *P* |
| --- | --- | --- | --- | --- | --- |
| Block | 1 | 0.66 | 0.662 | 0.034 | 0.856 |
| Error | 14 | 271.29 | 19.378 |  |  |
| Total | 15 | 271.95 | 20.04 |  |  |

**S3 Table. Least Mean Squares (+/– Standard Error) for ecosystem measurements: Chlorophyll-A (ug/cm2), AFDM (g/cm2) and Ammonium (ug/L)**

| Fish Treatment | Substrate | Chlorophyll-a  LSM(SE) | AFDM  LSM(SE) | Ammonium  LSM(SE) |
| --- | --- | --- | --- | --- |
| Control |  | 0.613 (0.2045) | 0.178 (0.023) | 292.675 (38.683) |
| HP | Gravel | 0.650 (0.2045) | 0.175 (0.023) | 241.410 (38.683) |
| LP |  | 0.738 (0.2045) | 0.142 (0.023) | 259.363 (38.683) |
| Control |  | 0.238 (0.2045) | 0.150 (0.023) | 356.150 (38.683) |
| HP | None | 0.275 (0.2045) | 0.147 (0.023) | 304.885 (38.683) |
| LP |  | 0.363 (0.2045) | 0.114 (0.024) | 322.839 (38.683) |

**S4 Table. The effects of fish (no-fish control, high predation (HP), low predation (LP)) and substrate (gravel, no gravel) on ecosystem response variables.**

| **Variable** | **Fish** | **Substrate** |
| --- | --- | --- |
| Ammonium | 0.64 _2,17_ | 2.84 _1,17_ |
| Chlorophyll-a | 0.16 _2,17_ | 4.17 _1,17_ $ |
| AFDM | 1.25 _2,16_ | 2.21 _1,16_ |
| Insect Abundance | 2.51 _2,17_ | 1.54 _1,17_ |
| Midge Abundance^a^ | 0.31 _2,15_ | 0.80 _1,15_ |
| Mosquito Abundance | 21.26 _2,17_ *** | 0.002 _1,16_ |
| Mayfly Abundance | 1.02 _2,17_ | 1.51 _1,17_ |

Main entries are F-ratios, degrees of freedom are listed in superscript. These values were obtained from linear mixed modeling as outlined in the text. Statistical significance is noted at the following levels: $ is p <0.10, * is p < 0.05, ** is p < 0.01, and *** is p < 0.001.

^a^This analysis had a significant interaction between fish and substrate (F-ratio 5.214_2,15_ **).

**S5 Table. Least Mean Squares (+/– Standard Error) for community composition: species richness (number of distinct taxa), Shannon-Weiner diversity index, and evenness.**

| Fish Treatment | Substrate | Richness  LSM(SE) | Diversity  LSM(SE) | Evenness  LSM(SE) |
| --- | --- | --- | --- | --- |
| Control |  | 12.29 (0.688) | 1.139 (0.133) | 0.447 (0.058) |
| HP | Gravel | 9.29 (0.688) | 0.885 (0.133) | 0.397 (0.058) |
| LP |  | 9.17 (0.688) | 0.889 (0.133) | 0.405 (0.058) |
| Control |  | 10.71 (0.688) | 1.193 (0.133) | 0.512 (0.058) |
| HP | None | 7.71 (0.688) | 0.939 (0.133) | 0.463 (0.058) |
| LP |  | 7.58 (0.688) | 0.943 (0.133) | 0.471 (0.058) |

**S6 Table. Raw means (+/­– Standard Error) for mg of insect biomass and abundance for total insects collected from the mesocosms, midges, mayflies and mosquitoes.**

| Insect Group | Fish Treatment | Substrate | Biomass  Mean (SE) | Abundance  Mean (SE) |
| --- | --- | --- | --- | --- |
| Total Insects | Control | Gravel | 25.795 (6.137) | 695 (91.2) |
|  |  | None | 44.353 (13.508) | 1308 (467) |
|  | HP | Gravel | 20.843 (2.833) | 656 (105) |
|  |  | None | 42.127 (8.973) | 675 (62.3) |
|  | LP | Gravel | 19.564 (2.824) | 728 (143) |
|  |  | None | 32.104 (9.385) | 658 (68.4) |
| Midges | Control | Gravel | 12.324 (4.623) | 417 (12.1) |
|  |  | None | 28.962 (6.530) | 778 (6.36) |
|  | HP | Gravel | 17.558 (3.350) | 585 (7.07) |
|  |  | None | 35.361 (9.246) | 506 (3.20) |
|  | LP | Gravel | 16.564 (1.716) | 592 (3.94) |
|  |  | None | 24.094 (5.369) | 488 (4.84) |
| Mayflies | Control | Gravel | 10.030 (9.814) | 121 (95.9) |
|  |  | None | 2.207 (1.342) | 95.8 (61.4) |
|  | HP | Gravel | 0.255 (0.246) | 7.25 (3.04) |
|  |  | None | 6.196 (3.300) | 104 (46.5) |
|  | LP | Gravel | 1.031 (0.373) | 50.8 (37.5) |
|  |  | None | 0.634 (0.567) | 102 (51.1) |
| Mosquitos | Control | Gravel | 3.121 (0.597) | 128 (18.2) |
|  |  | None | 2.558 (0.872) | 392 (290) |
|  | HP | Gravel | 0.495 (0.222) | 24.3 (9.14) |
|  |  | None | 0.378 (0.105) | 34.5 (8.76) |
|  | LP | Gravel | 0.039 (0.022) | 8.50 (4.84) |
|  |  | None | 0.042 (0.041) | 4.00 (3.08) |

**S7 Table. Least Square Means (+/– Standard Error) for mg of insect biomass and abundance (both log+1 transformed) for total insects collected from the mesocosms, midges, mayflies and mosquitoes.**

| Insect Group | Fish Treatment | Substrate | Biomass  LSM (SE) | Abundance  LSM (SE) |
| --- | --- | --- | --- | --- |
| Total Insects | Control | Gravel | 3.167 (0.196) | 6.723 (0.148) |
|  |  | None | 3.674 (0.196) | 6.892 (0.148) |
|  | HP | Gravel | 3.084 (0.196) | 6.404 (0.148) |
|  |  | None | 3.592 (0.196) | 6.568 (0.148) |
|  | LP | Gravel | 2.879 (0.196) | 6.424 (0.148) |
|  |  | None | 3.387 (0.196) | 6.589 (0.148) |
| Midges | Control | Gravel | 13.647 (5.075) | 417.25 (99.4) |
|  |  | None | 27.638 (5.075) | 585.25 (99.4) |
|  | HP | Gravel | 19.465 (5.075) | 592.25 (99.4) |
|  |  | None | 33.455 (5.075) | 777.75 (99.4) |
|  | LP | Gravel | 13.333 (5.075) | 506.25 (99.4) |
|  |  | None | 27.324 (5.075) | 488.25 (99.4) |
| Mayflies | Control | Gravel | 7.015 (3.966) | 4.90 (0.450) |
|  |  | None | 6.374 (3.966) | 4.88 (0.450) |
|  | HP | Gravel | 4.014 (3.966) | 3.28 (0.450) |
|  |  | None | 3.373 (3.966) | 3.26 (0.450) |
|  | LP | Gravel | 1.268 (3.966) | 1.31 (0.450) |
|  |  | None | 0.626 (3.966) | 1.29 (0.450) |
| Mosquitos | Control | Gravel | 0.580 (0.0429) | 4.896 (0.450) |
|  |  | None | 0.546 (0.0459) | 4.878 (0.450) |
|  | HP | Gravel | 0.166 (0.0422) | 3.276 (0.450) |
|  |  | None | 0.132 (0.0422) | 3.258 (0.450) |
|  | LP | Gravel | 0.0335 (0.0422) | 1.309 (0.450) |
|  |  | None | -0.000343 (0.0422) | 1.291 (0.450) |

**S8 Table. Results of Fisher’s exact tests comparing presence/absence of insects in HP and LP guppy diet.**

| Prey | P-value | Odds Ratio |
| --- | --- | --- |
| All Insects | 0.038 | 3.204 |
| Chironomids | 0.112 | 2.452 |
| Mosquitoes | 0.285 | 2.506 |
