# Supplementary material for: Implications of guppy (Poecilia reticulata) life‐history phenotype for mosquito control
Source: Ecol Evol. 2017 Apr 1;7(10):3324–34. doi: 10.1002/ece3.2666 (PMC5433973; doi:10.1002/ece3.2666)
Supplement: Supplementary file 2 [file ECE3-7-3324-s002.docx]

**Supplementary Information**

**Appendix 2 – Insect Length-Mass Relationships**

Table 1. Equations and associated R-squared values for insect length-mass relationships for both dry mass (DM) and ash-free dry mass (AFDM). Average percent ash is also present for each taxa, with standard error in brackets.

| **Order** | **Taxa** | **N** | **Metric** | **Equation** | **R_2_** | **% Ash** |
| --- | --- | --- | --- | --- | --- | --- |
| Diptera | Ceratopogonidae *Forcipomyia* | 24 | DM | y = 0.0017x^2.2551^ | 0.668 | 0.273 (0.023) |
|  |  | 24 | AFDM | y = 0.0008x^2.7984^ | 0.760 |  |
|  | Chironomidae Tanypodinae | 43 | DM | y = 0.0034x^1.9045^ | 0.829 | 0.270 (0.022) |
|  |  | 43 | AFDM | y = 0.0015x^2.2856^ | 0.937 |  |
|  | Chironomidae non-Tanypodinae | 55 | DM | y = 0.0009x^2.2856^ | 0.951 | 0.270 (0.024) |
|  |  | 53 | AFDM | y = 0.0002x^2.8744^ | 0.940 |  |
|  | Chironomidae (pupae) | 29 | DM | y = 0.002x^2.5512^ | 0.981 | 0.098 (0.013) |
|  |  | 29 | AFDM | y = 0.0017x^2.5735^ | 0.980 |  |
|  | Culicidae Anopheles | 37 | DM | y = 0.001x^2.8734^ | 0.941 | 0.306 (0.027) |
|  |  | 37 | AFDM | y = 0.0001x^3.9913^ | 0.944 |  |
|  | Culicidae Culex | 34 | DM | y = 0.0011x^2.4969^ | 0.925 | 0.241 (0.020) |
|  |  | 33 | AFDM | y = 0.0005x^2.8063^ | 0.936 |  |
|  | Culicidae Culiseta | 26 | DM | y = 0.0003x^3.395^ | 0.951 | 0.173 (0.011) |
|  |  | 26 | AFDM | y = 0.0002x^3.5075^ | 0.953 |  |
|  | Culicidae Wyeomiya | 24 | DM | y = 0.0021x^2.1189^ | 0.906 | 0.387 (0.026) |
|  |  | 24 | AFDM | y = 0.0006x^2.5665^ | 0.889 |  |
| Ephemeroptera | Baetidae Baetodes | 28 | DM | y = 0.0021x^2.4495^ | 0.886 | 0.226 (0.019) |
|  |  | 28 | AFDM | y = 0.0014x^2.5881^ | 0.821 |  |
|  | Caenidae Caenis | 35 | DM | y = 0.0041x^2.2651^ | 0.953 | 0.232 (0.010) |
|  |  | 34 | AFDM | y = 0.0032x^2.2666^ | 0.963 |  |
|  | Leptophlebiidae Thraulodes | 30 | DM | y = 0.0021x^3.1348^ | 0.961 | 0.159 (0.011) |
|  |  | 30 | AFDM | y = 0.0016x^3.2016^ | 0.970 |  |
| Thichoptera | Calamoceratidae Phylloicus | 21 | DM | y = 0.0006x^3.0605^ | 0.988 | 0.111 (0.013) |
|  |  | 22 | AFDM | y = 0.0004x^3.1753^ | 0.990 |  |
|  | Philopotamidae Chimarra | 13 | DM | y = 0.0011x^2.5463^ | 0.963 | 0.125 (0.014) |
|  |  | 13 | AFDM | y = 0.0008x^2.6585^ | 0.964 |  |
| Collembola | Collembola | 29 | DM | y = 0.0043x^2.5216^ | 0.999 | 0.127 (0.012) |
|  |  | 29 | AFDM | y = 0.0034x^2.6832^ | 0.998 |  |

Order Diptera (True Flies)


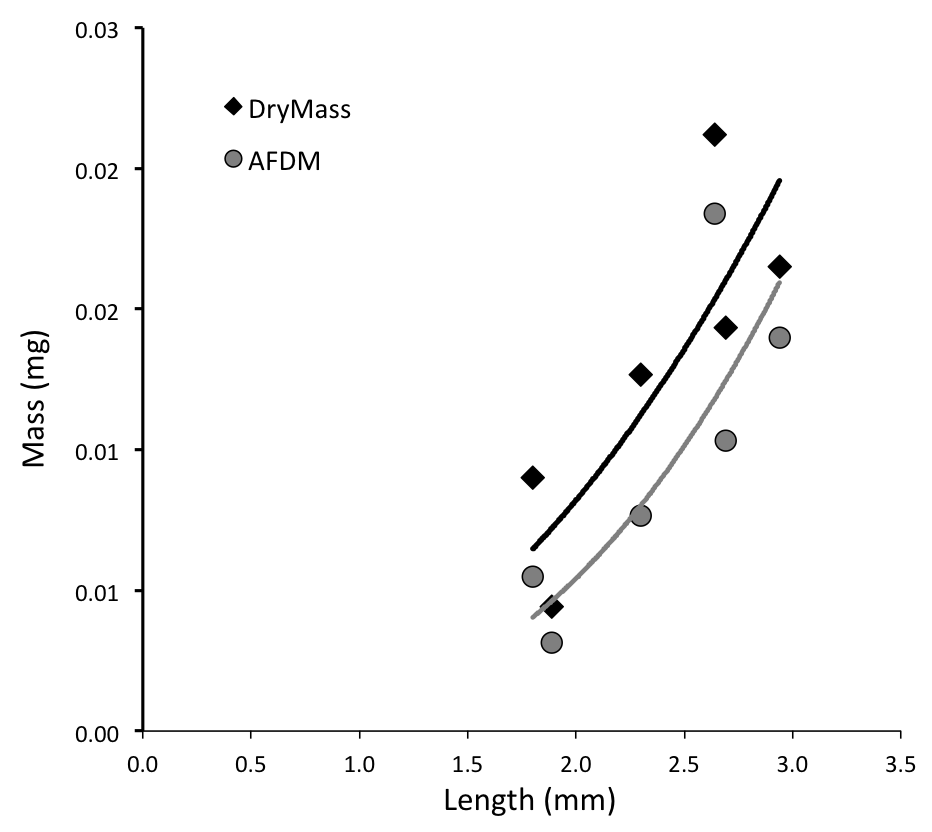


Fig 2. Length-mass relationship for Ceratopogonidae *Forcipomyia.* Black diamonds show insect dry mass. Grey circles are ash-free dry mass.


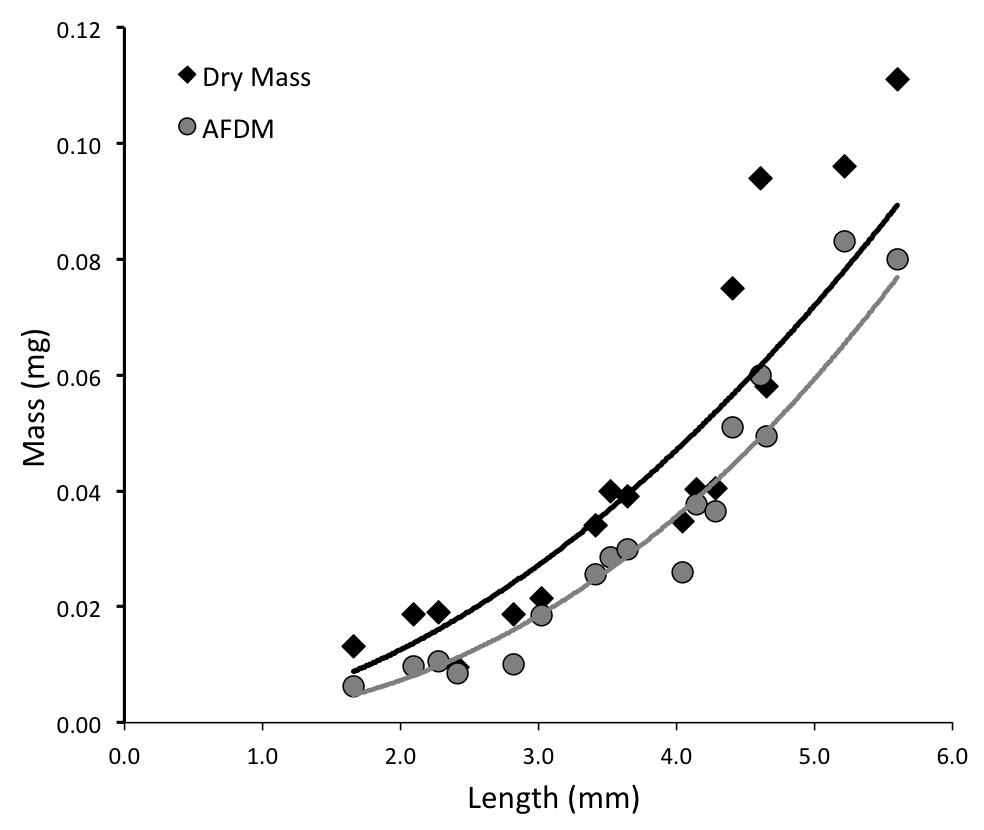


Figure 3. Length-mass relationship for Chironomidae Tanypodinae subfamily*.* Black diamonds show insect dry mass. Grey circles are ash-free dry mass.


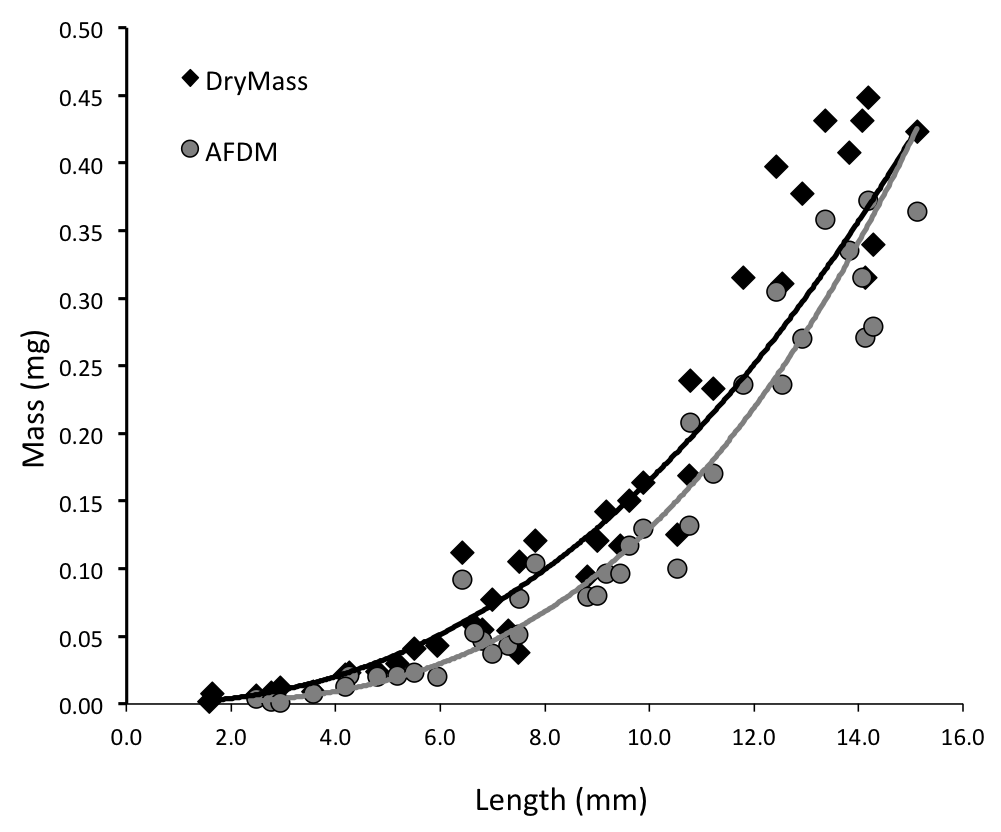


Figure 4. Length-mass relationship for Chironomidae non-Tanypodinae subfamily*.* Black diamonds show insect dry mass. Grey circles are ash-free dry mass.


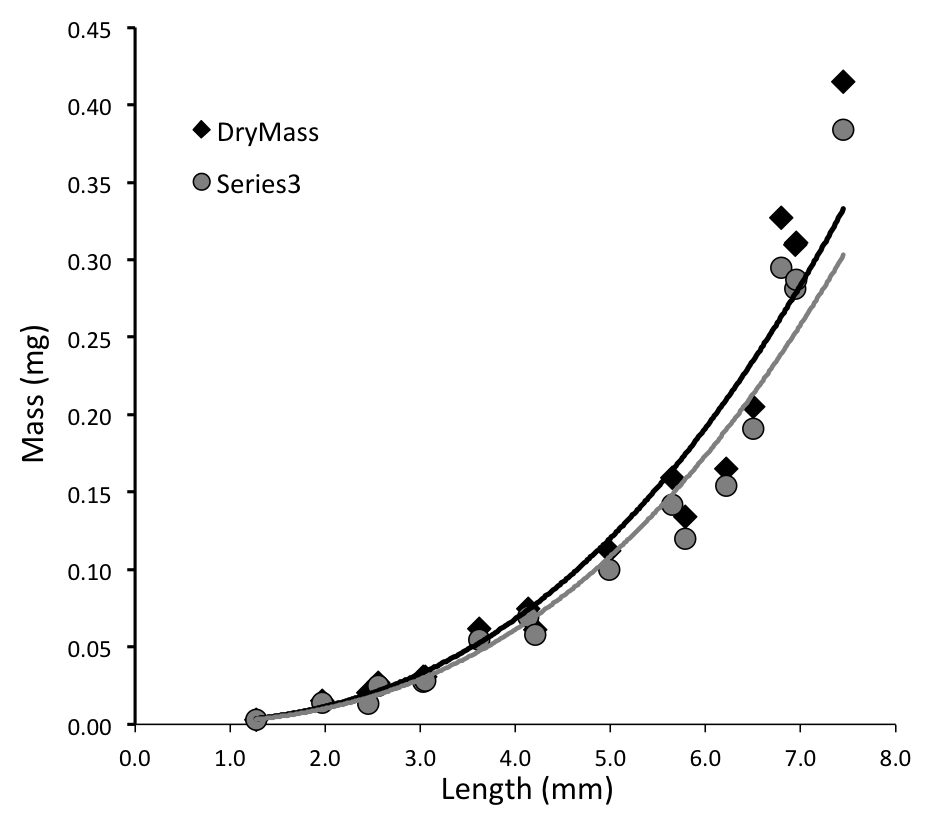


Figure 5. Length-mass relationship for Chironomid pupae*.* Black diamonds show insect dry mass. Grey circles are ash-free dry mass.


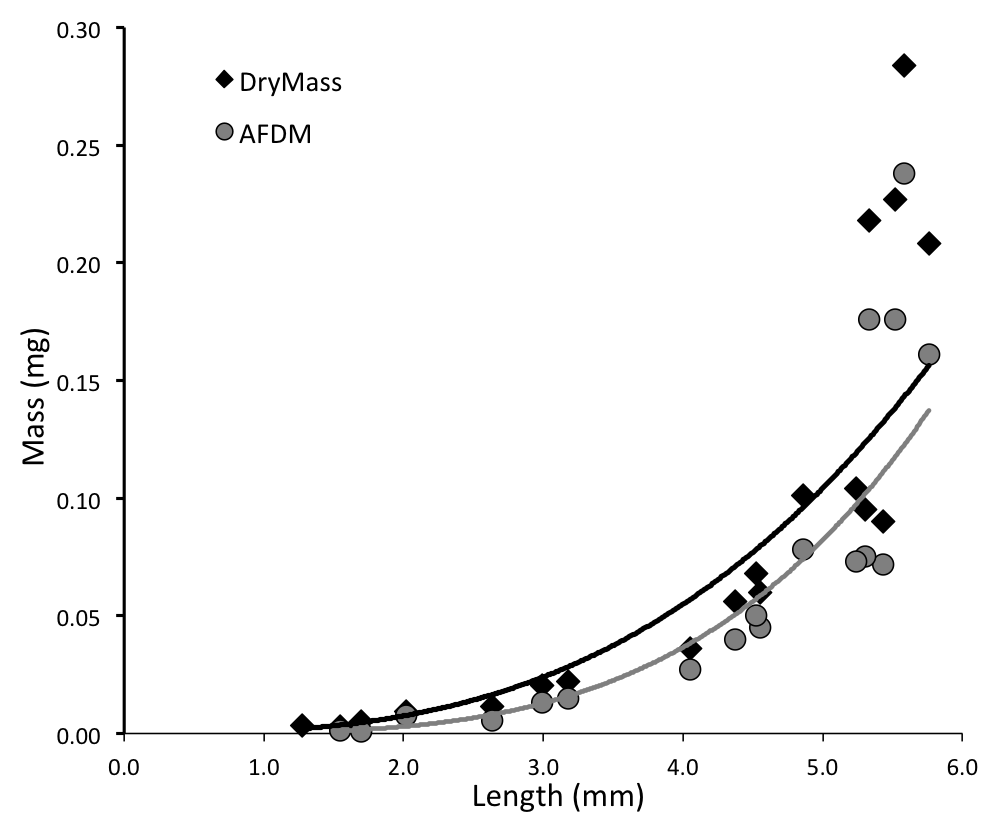


Figure 6. Length-mass relationship for Culicidae *Anopheles.* Black diamonds show insect dry mass. Grey circles are ash-free dry mass.


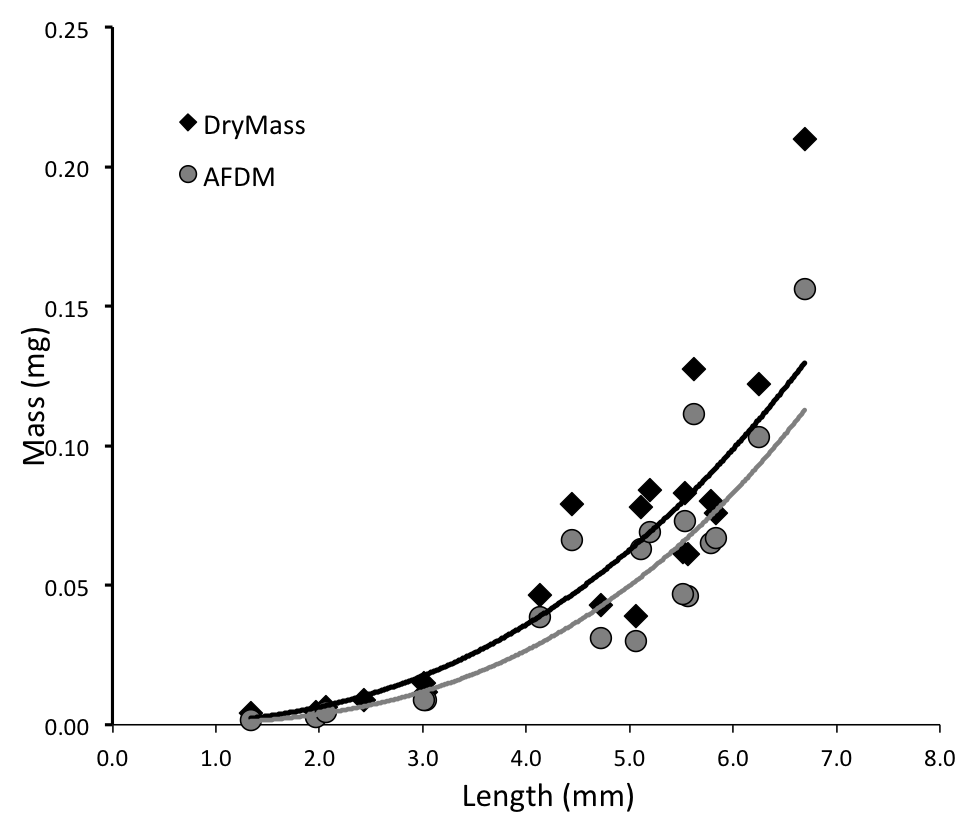


Figure 7. Length-mass relationship for Culicidae *Culex.* Black diamonds show insect dry mass. Grey circles are ash-free dry mass.


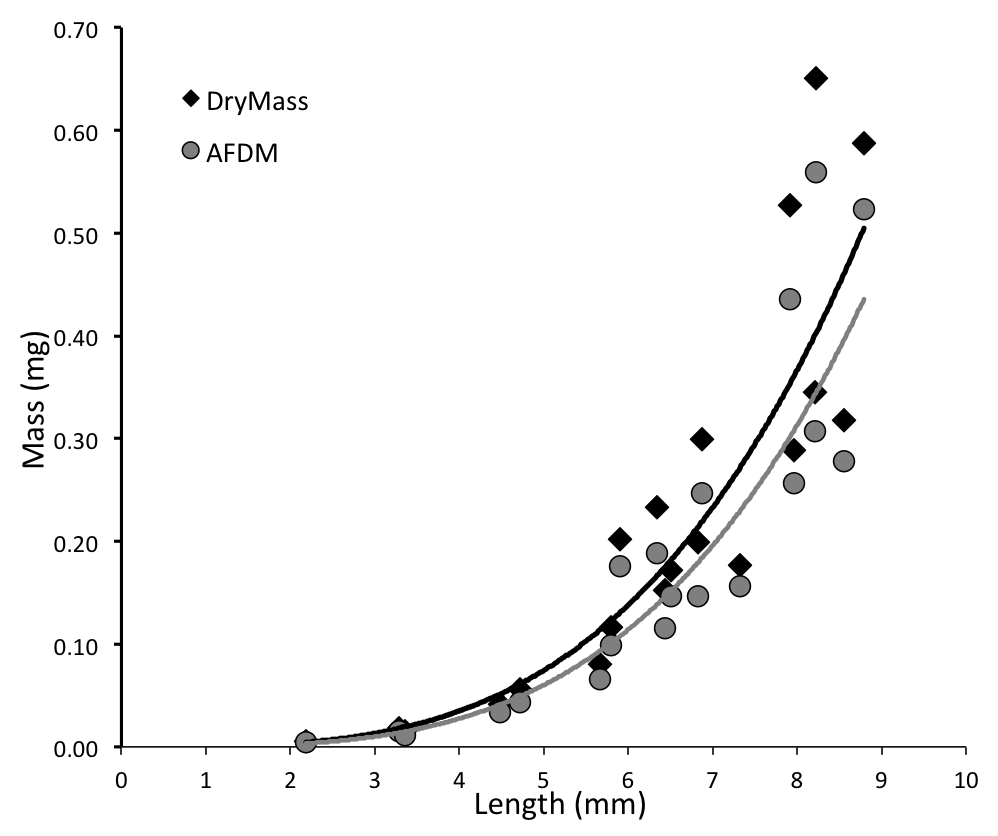


Figure 8. Length-mass relationship for Culicidae *Culiseta.* Blue diamonds show insect dry mass. Red squares are estimated dry ash-free dry mass. Green circles are actual ash-free dry mass.


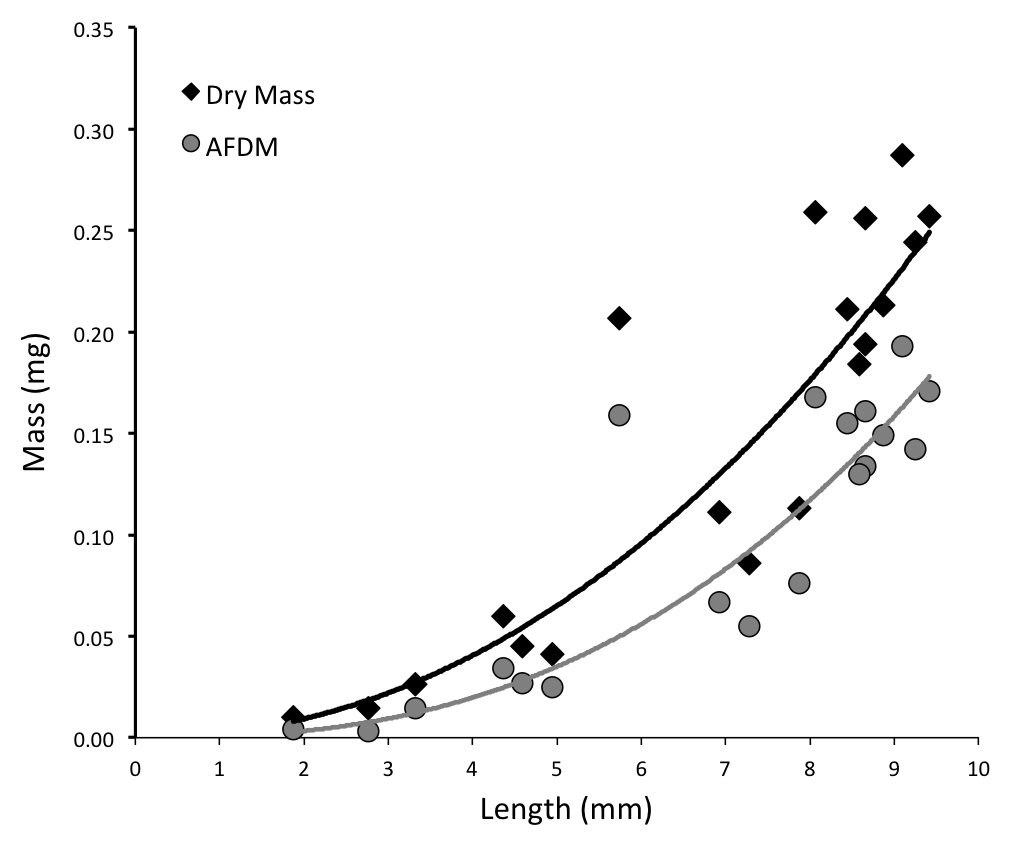


Figure 9. Length-mass relationship for Culicidae *Wyeomyia.* Blue diamonds show insect dry mass. Red squares are estimated dry ash-free dry mass. Green circles are actual ash-free dry mass.

Order Ephemeroptera (Mayflies)


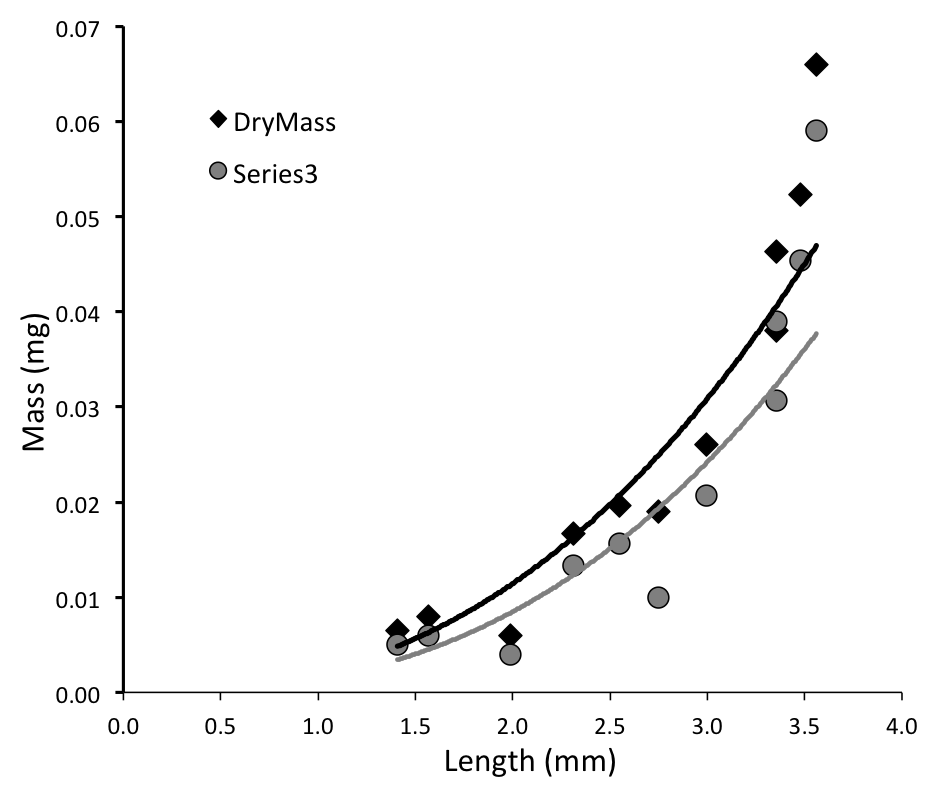


Fig 10. Length-mass relationship for Baetidae *Baetodes.* Black diamonds show insect dry mass. Grey circles are ash-free dry mass.


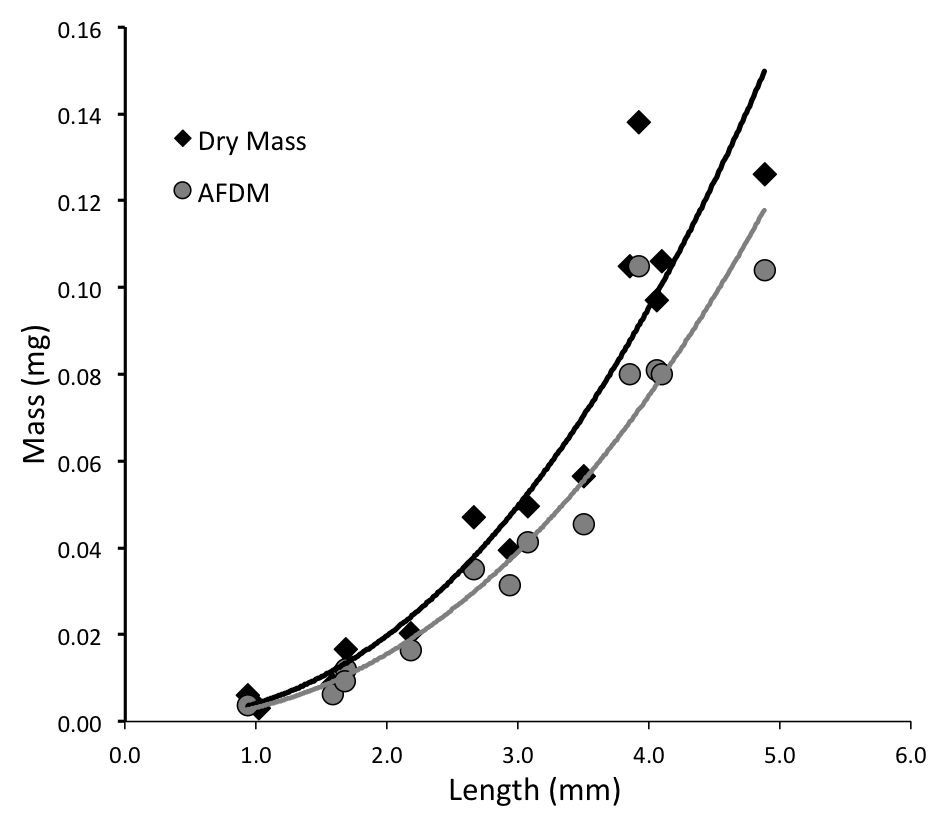


Fig 11. Length-mass relationship for Caenidae *Caenis spp.* Black diamonds show insect dry mass. Grey circles are ash-free dry mass.


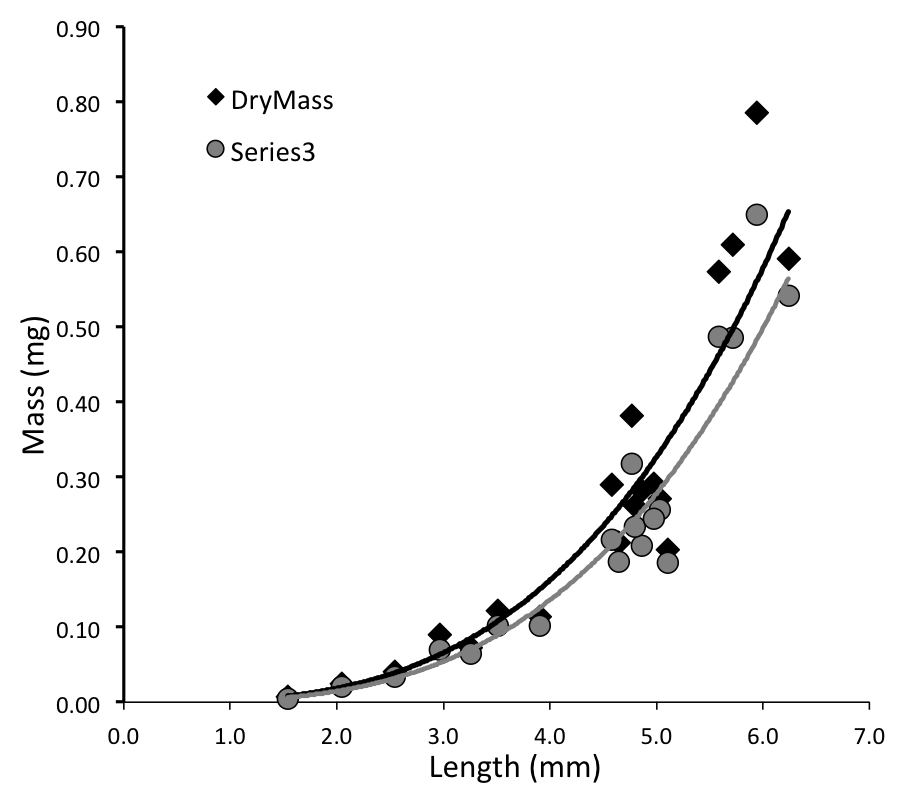


Fig 12. Length-mass relationship for Leptophlebiidae *Thraulodes.* Black diamonds show insect dry mass. Grey circles are ash-free dry mass.

Order Trichoptera (Caddisflies)


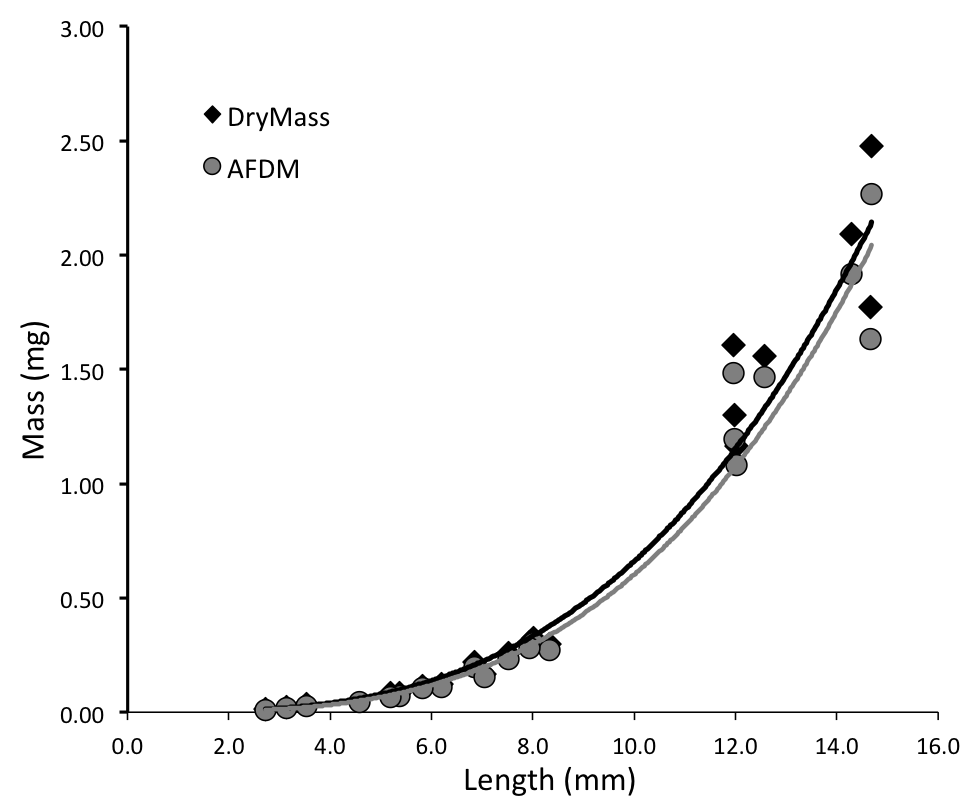


Fig 13. Length-mass relationship for Calamoceratidae *Phylloicus.* Black diamonds show insect dry mass. Grey circles are ash-free dry mass.


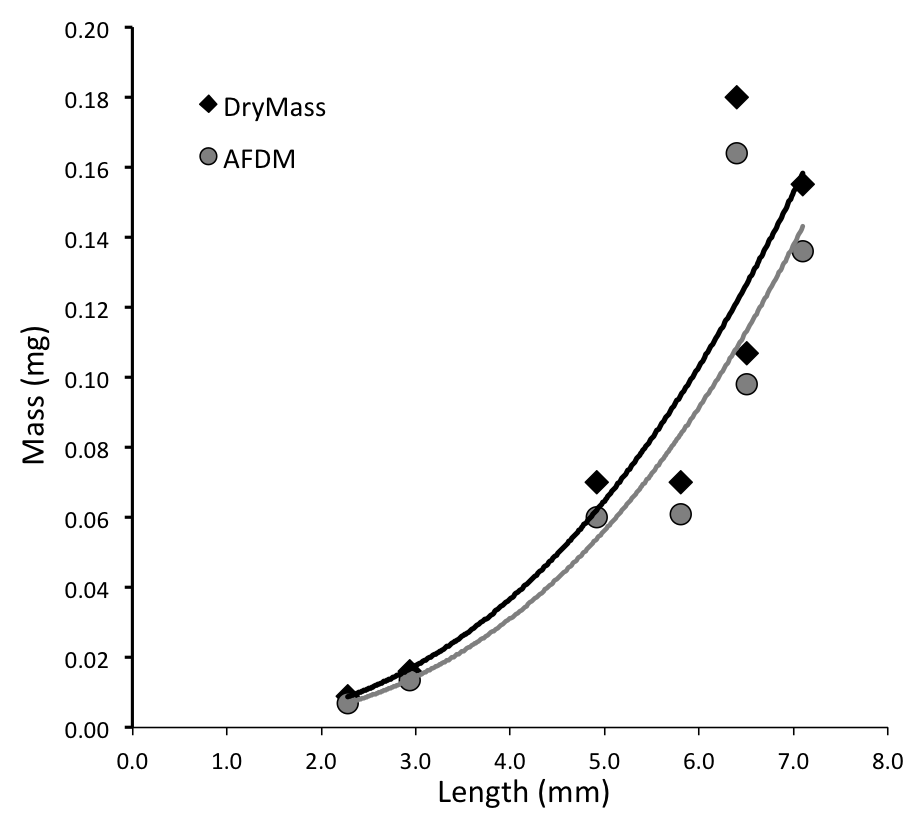


Fig 14. Length-mass relationship for Philopotamidae *Chimarra.* Black diamonds show insect dry mass. Grey circles are ash-free dry mass.

Order Collembola


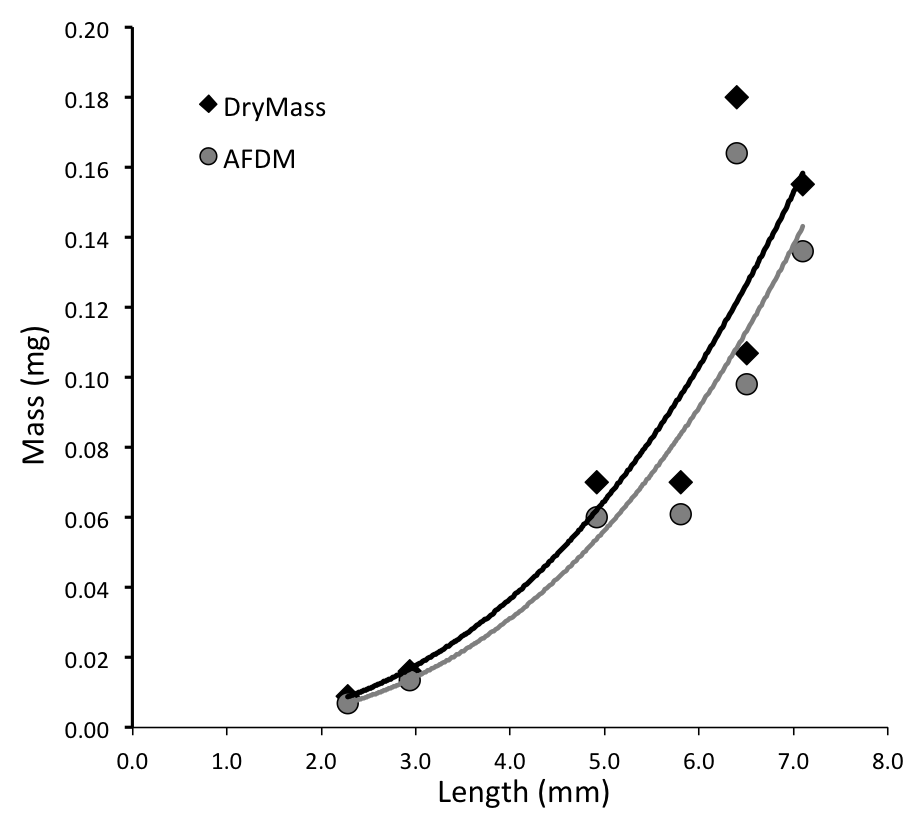


Figure 15. Length-mass relationship for Collembola*.* Black diamonds show insect dry mass. Grey circles are ash-free dry mass.
